# Use of Potent Topical Corticosteroids (TCS) for Hypergranulation Tissue (HGT) in Pediatric Patients

**DOI:** 10.7759/cureus.28304

**Published:** 2022-08-23

**Authors:** Shae Margulies, Tyler Marion, Sami K Saikaly

**Affiliations:** 1 Department of Dermatology, University of Florida College of Medicine, Gainesville, USA

**Keywords:** granulation tissue, wound healing, topical corticosteroids, pediatric dermatology, hypergranulation tissue

## Abstract

Hypergranulation is a common complication of wound healing that dermatologists encounter and is characterized by excess granulation tissue, which results in delayed healing and reepithelialization. Though many treatment options have been presented in the literature, less invasive and irritating treatment regimens are often preferred in the pediatric population. Here we present a case of a 14-year-old female with hypergranulation tissue (HGT) of the scalp that was successfully treated with topical corticosteroids (TCS). Additionally, a literature review was conducted to determine the prevalence of topical corticosteroid use for HGT in the pediatric population. Although not first line, TCS should be considered as a non-invasive and painless treatment for HGT in the pediatric population.

## Introduction

Hypergranulation is characterized by excess granulation tissue, which results in delayed wound healing and reepithelialization. Hypergranulation tissue (HGT) frequently presents as friable red tissue that extends above and beyond the original surface of the wound [1]. Various treatment options have been presented in the literature, such as silver nitrate and surgical debridement, without a distinct optimal choice [2]. However, less invasive and less irritating treatment regimens are preferred in the pediatric population. Here we present a case reporting the successful use of high-potency topical corticosteroids (TCS) for the treatment of HGT in a pediatric patient.

## Case presentation

A 14-year-old previously healthy female presented to the clinic accompanied by her mother for complaints of a scalp burn. Six weeks prior, the patient had dyed her hair with an expired hair product resulting in scalp pain and irritation. She visited the emergency department (ED) where she presented with an erythematous, scaly scalp erosion. She was prescribed cephalexin and silver sulfadiazine every other day and then daily for 10 days. Two weeks after initially healing, she developed worsening pain, scaling, and discharge. She returned to the ED and was given a 10-day course of clindamycin 300 mg four times daily for possible infection. She was then referred to pediatric dermatology.

**Figure 1:**
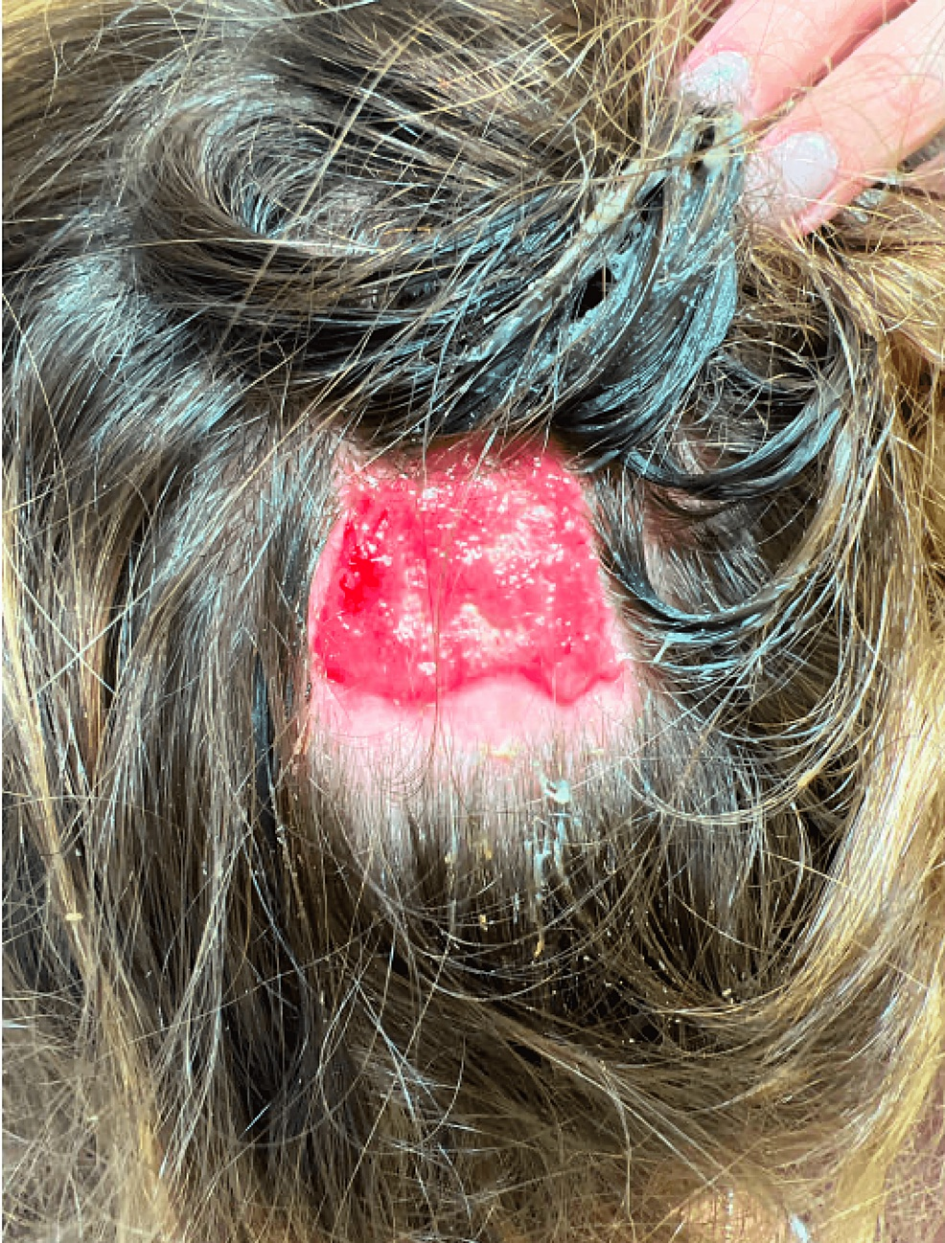
3.5 x 3.0 cm red and friable eroded plaque on vertex scalp at clinic visit

**Figure 2:**
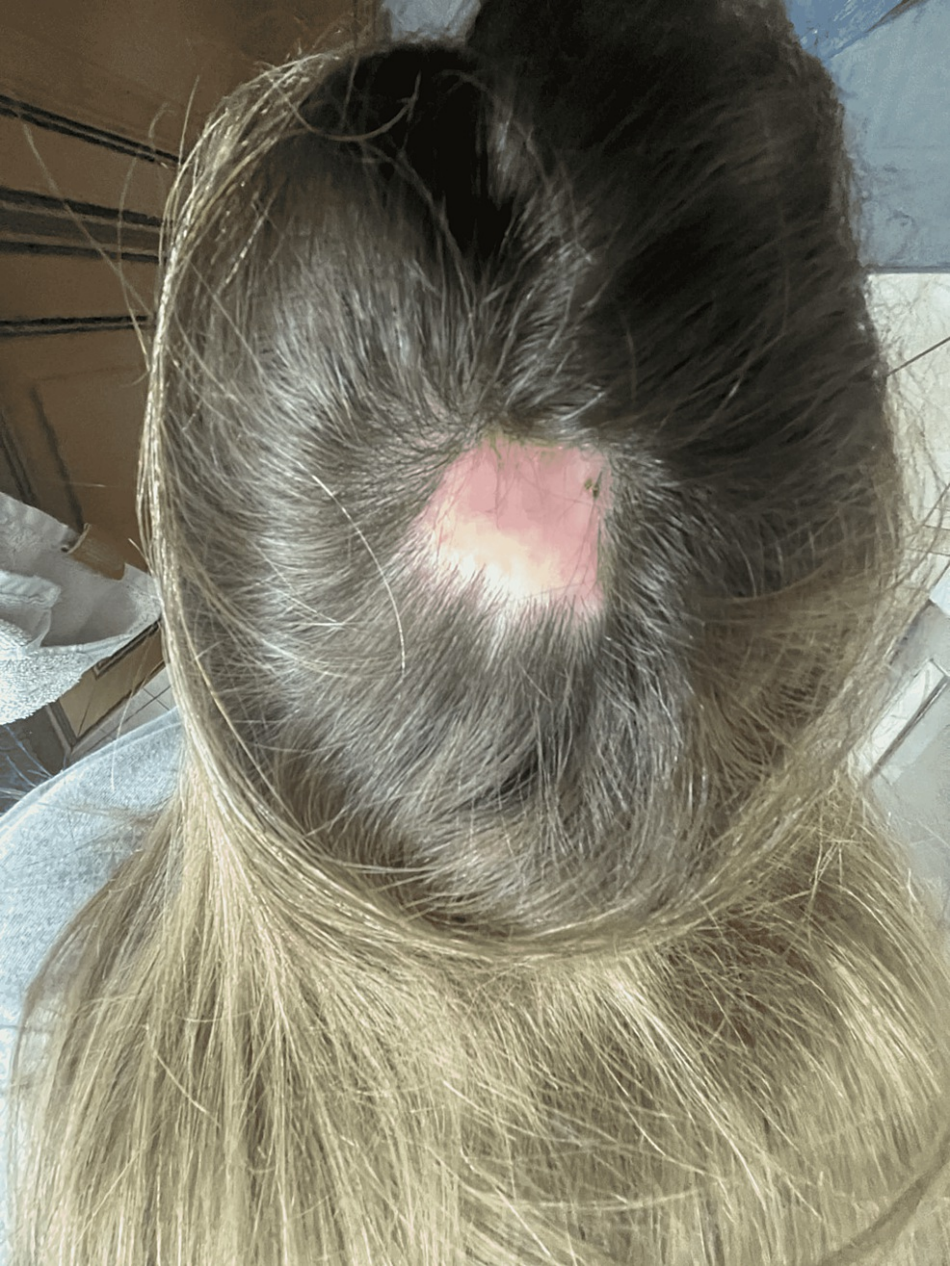
Complete resolution of hypergranulation tissue (HGT) after clobetasol use

During clinic follow up, the patient stated that the lesion had not adequately improved despite treatment. On examination, a 3.5 x 3.0 cm red, friable eroded plaque was noted on the vertex scalp (Figure 1). She was diagnosed with HGT and was prescribed clobetasol 0.05% ointment to apply topically twice daily. After six weeks of treatment, she had complete resolution (Figure 2). 

Given the characteristic physical examination and clinical history, the diagnosis of HGT was highly suspected over less likely differential diagnoses of pyogenic granuloma and a cutaneous malignancy. The patient's parent deferred a biopsy in place of empiric treatment and a follow-up six weeks later to reassess clinical progress. In cases where the diagnosis is unclear or empiric treatment fails, however, a biopsy should be performed to confirm the diagnosis. If there was clinical suspicion of a potentially harmful diagnosis, such as malignancy or infection, a biopsy would have been recommended. Additionally, if the patient was not clinically improving at her six-week follow up, biopsy would be the next step. 

## Discussion

HGT is characterized by overgrowth of fibroblasts and endothelial cells that impedes healing by preventing the migration of epithelial cells across the wound. It is associated with an increased risk of infection [1]. Scalp surgical wounds, in particular, have increased susceptibility to developing HGT [3,4].

Traditional treatments for HGT include silver nitrate, TCS, surgical debridement, and pulsed dye laser [2]. Less commonly, topical timolol has been used [5]. Silver nitrate, while usually first line, is often ineffective and painful [2,6]. Pulsed dye laser treatments target the increased vascular supply of HGT, and are often effective in refractory cases [4]. However, multiple treatments are often required to achieve resolution, which can be time consuming and costly. Although not considered first line, the use of TCS has shown promise as an effective and non-painful option [6,7]. Reported successful treatments of pediatric HGT with TCS are summarized in Table 1 [3]. A recent review of the literature compiled over 30 cases of both adult and pediatric patients with HGT, showing resolution of lesions with various TCS [6]. 

**Table 1:** Reported cases of topical corticosteroids (TCS) for hypergranulation tissue (HGT) in pediatric patients Summary of cases reported in [3]

Patient Age	Mechanism of Injury	Location	Treatment	Outcome
3 yrs	Shave biopsy	Parietal scalp	Clobetasol ointment 0.05%	Almost complete resolution at two weeks
8 yrs	Excision	Parietal scalp	Fluocinonide ointment 0.05%	Significant improvement at two weeks, complete resolution at two months
10 yrs	Crush injury	Thumb	Fluocinonide ointment 0.05%	Complete resolution at two weeks

When treating the pediatric population, great consideration should be made to select therapies that are effective while also causing little discomfort. This principle is followed in other aspects of pediatric dermatology, such as in the treatment of molluscum contagiosum. While curettage of molluscum contagiosum lesions is an effective treatment method, this can cause significant fear and pain in pediatric patients, and is therefore avoided [8]. Therapies such as silver nitrate, surgical debridement, and lasers can be painful and traumatizing to children, and can leave them and their caregivers dissatisfied with their care. In contrast, TCS are not painful and can be applied at home by a caregiver to minimize trauma and fear in the child. While a majority of reported cases describe the use of potent TCS in the treatment of HGT, low potency TCS have also shown good efficacy [9]. Thus, it is recommended that a trial with low potency TCS is performed prior to progressing to more potent options. Further, no local or systemic side effects of TCS treatment for HGT have been reported in the literature [6,9].

## Conclusions

In conclusion, high potency TCS are an effective treatment for HGT and should be considered as a treatment option in the pediatric population. Special care should be taken when treating children to avoid discomfort while maximizing results. Unlike other standard treatments, TCS are painless and non-invasive, making them a more palatable option for children and their caretakers. This case report adds to the literature evidence of resolution of HGT tissue after use of clobetasol 0.05% ointment, without the occurrence of patient discomfort. Further studies may be warranted to compare long term outcomes and comparison of cosmetic results with other, more traditional treatment modalities.
